# The value of a digital management system for the medical records of patients with cerebral hemorrhage

**DOI:** 10.3389/fpubh.2023.973289

**Published:** 2023-03-07

**Authors:** Qin Zhang, Xiao-Dong Li

**Affiliations:** Department of Medical Records Statistics, Western Hospital of Beijing Chaoyang Hospital Affiliated to Capital Medical University, Beijing, China

**Keywords:** micro-digital imaging technology, digital management system, cerebral hemorrhage, medical record management, stroke

## Abstract

**Objective:**

The present study aimed to explore the value of a digital management system based on micro digital imaging technology for the medical records of patients with cerebral hemorrhage.

**Methods:**

A total of 540 patients with intracerebral hemorrhage diagnosed and treated in the hospital from November 2016 to November 2019 were selected as the subjects of this study. A digital management system based on micro digital imaging technology was used to establish how to handle the medical records of these patients.

**Results:**

The medical record management process of patients with cerebral hemorrhage comprises five modules: medical record editing, medical record querying, medical record borrowing management, statistical analysis, and system maintenance. The purpose of these is to manage and store digitalised medical records for patients, present the most useful information on the first page of the medical records, monitor the safety of the medical records, and enable real-time data sharing.

**Conclusion:**

Establishing a digital management system based on micro digital imaging technology for the medical records of patients with cerebral hemorrhage can effectively enable the preservation and use of these documents. It improves information management, such as data sharing, security, and the overall level of information. This is worth promoting.

## 1. Introduction

Cerebral hemorrhage is a common pathological type of stroke. In China, patients with cerebral hemorrhage account for about 25% of all stroke types, and their morbidity and mortality are high (1). The clinical manifestations of patients with intracerebral hemorrhage are aphasia, hemiplegia, disturbance of consciousness, and other neurological damage. The condition is dangerous and poses a serious threat to a patient's life and health (2). Surgery and targeted treatment are the primary methods used to treat cerebral hemorrhage, but the clinical outcome for patients is still not ideal because of the limitation of the clinical treatment plans (3). Therefore, it is important to actively evaluate and predict the clinical outcome for patients with cerebral hemorrhage and take timely targeted measures to improve their prognosis.

Medical records refer to the files that record a patient's enquiry, examination, diagnosis, and treatment, and they can provide guidance for clinicians in developing a sound diagnosis and treatment plan (4). For a long time, as the only preservation of medical records, paper has caused many problems in medical record management in hospitals. For example, it is difficult to consult and borrow medical records if storage space is insufficient and the records are disorganized. Additionally, there is a security issue regarding preservation. Mildew, moths, illegible handwriting, natural loss, and other phenomena may cause irreparable losses. A more critical problem is that it is difficult to use medical record information on paper effectively. With the expansion of the hospital, the number of medical records and the demand for their use are increasing, resulting in an increasingly prominent contradiction between safe storage and full utilization. The traditional way of keeping medical records has restricted the development of modern hospital medical record management (5).

With the rapid development of internet technology and the continuous improvement in managing information, digital medical record management has become an inevitable trend in hospitals (6). With this, the digital medical record is the important basic content, and the management system provides data statistics, querying, analysis, and summary functions (7). A digital management system based on micro digital image technology is a means of combining these two facets with medical record theory to establish a new mode of digital, information-based, and heterogeneous micro imaging medical record historical information management (8).

The micro digital image technology system comprises photographic density; image-resolving power; and washing, processing, and replication technology, which can form micro images and digital images simultaneously through one-time acquisition. It does not need to manually process the conversion between micro images and digital images to achieve a remote heterogeneous backup of medical records (9). The micro image has legal status and can be permanently saved, which effectively reduces the risk of medical record loss. The digital image can be stored in the medical record management system for effective electronic management to reduce access to the original paper medical record. In addition, it can also reduce the cost of managing paper medical records (10).

The present study collected the relevant medical records of patients diagnosed with intracerebral hemorrhage between November 2016 and November 2019 and built a digital management system based on micro digital image technology to manage these medical records.

## 2. Data and methods

### 2.1. Data source

A total of 540 patients with intracerebral hemorrhage diagnosed and treated in the hospital from November 2016 to November 2019 were selected as the subjects of this study. Relevant medical records were collected to understand the practice and methods of medical record management. In addition, through testing Wanfang, the Chinese Scientific Journals database, China National Knowledge Infrastructure, PubMed, and other domestic and foreign electronic databases, the research status, and development trends of hospital medical record management domestically and internationally were understood.

### 2.2. A digital management system based on micro digital image technology

Using the digital management system based on micro digital image technology, this study provides a model of how to handle medical record management for patients with cerebral hemorrhage. The key management contents of each stage are described below.

#### 2.2.1. Registration and storage of paper medical records

The medical records of patients with cerebral hemorrhage were completed according to the basic specification for writing medical records (wyzf [2010] No. 11) and the quality specification for filling data on the first page of inpatient medical records (Provisional) (gwbf YF [2016] No. 24). The process was: (1) a new medical record was created, and the patient's name, gender, age, home address, contact information, and other basic information were completed; and (2) according to the diagnosis and treatment, the patient's medical record was truthfully and normatively written.

#### 2.2.2. Completion of digital medical records

Using micro digital imaging technology, medical record data were scanned in digital form, including patient name, gender, age, date of birth, nationality, occupation, home address, contact information, marital status, family history of cerebral hemorrhage, disease diagnosis, drug allergy history, date of visit, medical history data, diagnostic information (including various imaging data), surgical records, relevant examination records, and treatment information. Scanning the bar code on the first page of the medical record stores the medical record in the database and indexes it for later retrieval by the management software. This overcomes the restrictions of paper medical records.

#### 2.2.3. Digital medical record printing

To avoid the loss of paper medical records, the digital medical records were printed after the patient left the hospital, except for the section with their signature. In this way, the management software replaced the traditional heavy task of externally copying the medical records, which was tedious and inefficient work with low satisfaction.

### 2.3. Connection between the digital management system and the hospital information management system

The digital medical record management system based on micro digital image technology was connected to the hospital information management system so doctors anywhere in the hospital could access authorized medical records from their workstations by verifying their username and password. To restrict sharing medical information, access to medical records beyond a doctor's authority could be applied for, and they could be accessed after approval by the medical record managers.

## 3. Results

### 3.1. Establishment of a medical record management process for patients with cerebral hemorrhage

**Figure 1:**
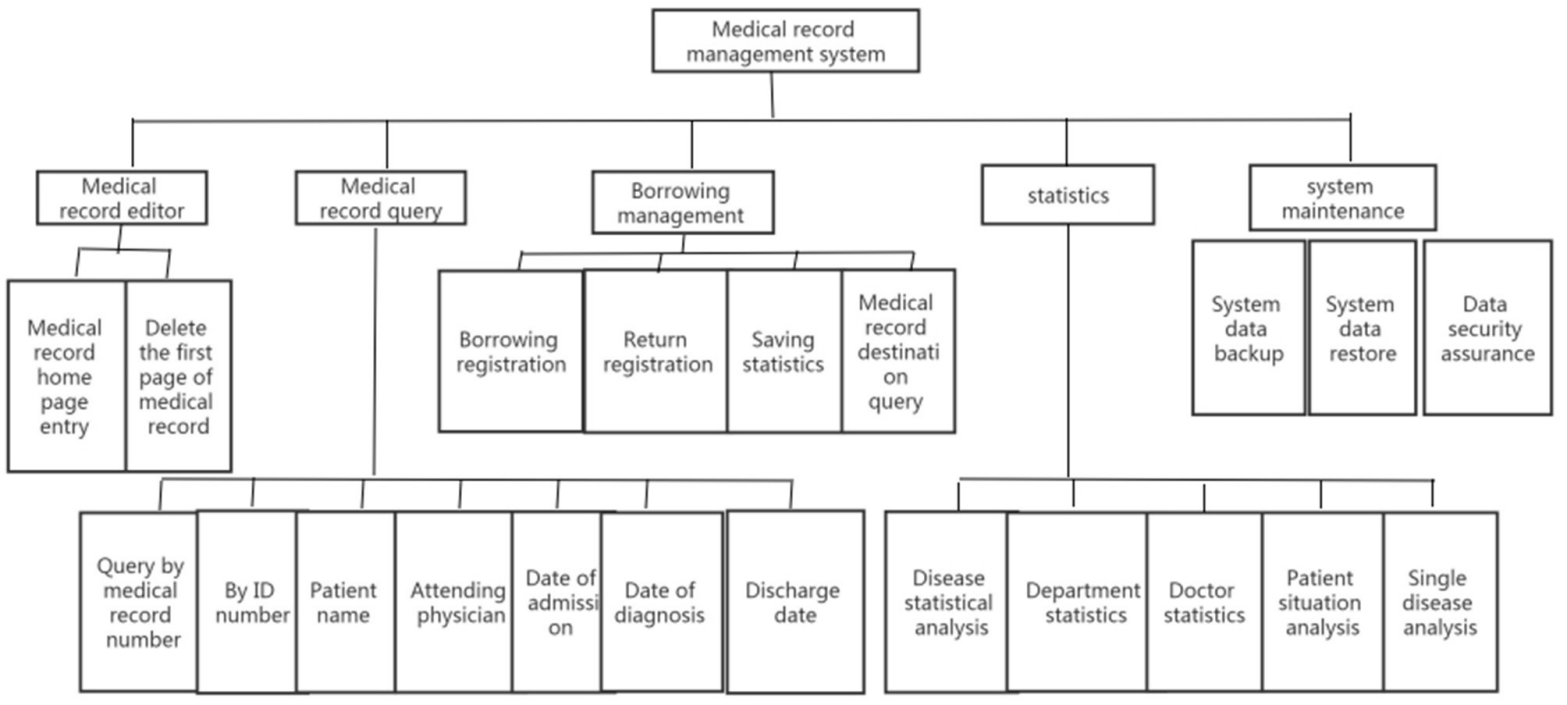
Flow chart of medical record management for patients with cerebral hemorrhage.

The medical record management process for patients with cerebral hemorrhage comprises five modules: medical record editing, medical record querying, medical record borrowing management, statistical analysis, and system maintenance. See Figure 1 for the detailed flow chart.

The medical record management home page enables querying and editing. It includes the patient's basic personal information, disease diagnosis, surgery, treatment results, medical record quality, and other information. The patient's name and medical record number can be used for querying, and the retrieval results show the information index of the medical record home page.

Medical record borrowing management includes registration, return registration, warehousing, and lending situation analysis.

The medical record statistics module allows various monthly, quarterly, and annual reports to be generated, which is helpful in the analysis of data related to cerebral hemorrhage requested by clinical and scientific research staff.

System maintenance involves ensuring system safety and maintaining its normal operation.

The overall medical record management system can collect, process, store, retrieve, and transmit the relevant information of patients with cerebral hemorrhage and provide valuable information to medical staff.

### 3.2. Establishment of a digital management system based on micro digital imaging technology to manage the medical records of patients with cerebral hemorrhage

Implementing digital management based on micro digital imaging technology can significantly reduce the storage space required for medical records. The micro digital imaging technology has a fast scanning speed and a one-to-one correspondence with the paper medical records, which can ensure that medical records are quickly stored and the required floor area of the storage warehouse is reduced. It also reduces the potential damage to the medical records caused by natural and human factors and ensures the integrity of the originals.

Additionally, it can effectively improve the efficiency of consulting the records. The digital management system includes a retrieval system and a printing system. Patients, family members, and medical staff can consult and print medical records on demand to share medical information. This can avoid unused paper medical records. Implementing a digital management system can effectively reduce errors from manual binding and sorting, save time searching for and storing paper records, overcome the problem of not locating medical records, and ensure the orderly progress of medical record management. It also enables effective backup of medical record data. Only some of the medical records in the subject hospital are digital medical records. There are still handwritten medical records, including nursing records and doctor consultation sheets, which are easily lost. Therefore, a digital management system allows the backup of medical record data and improves the current situation of medical record management.

**Figure 2:**
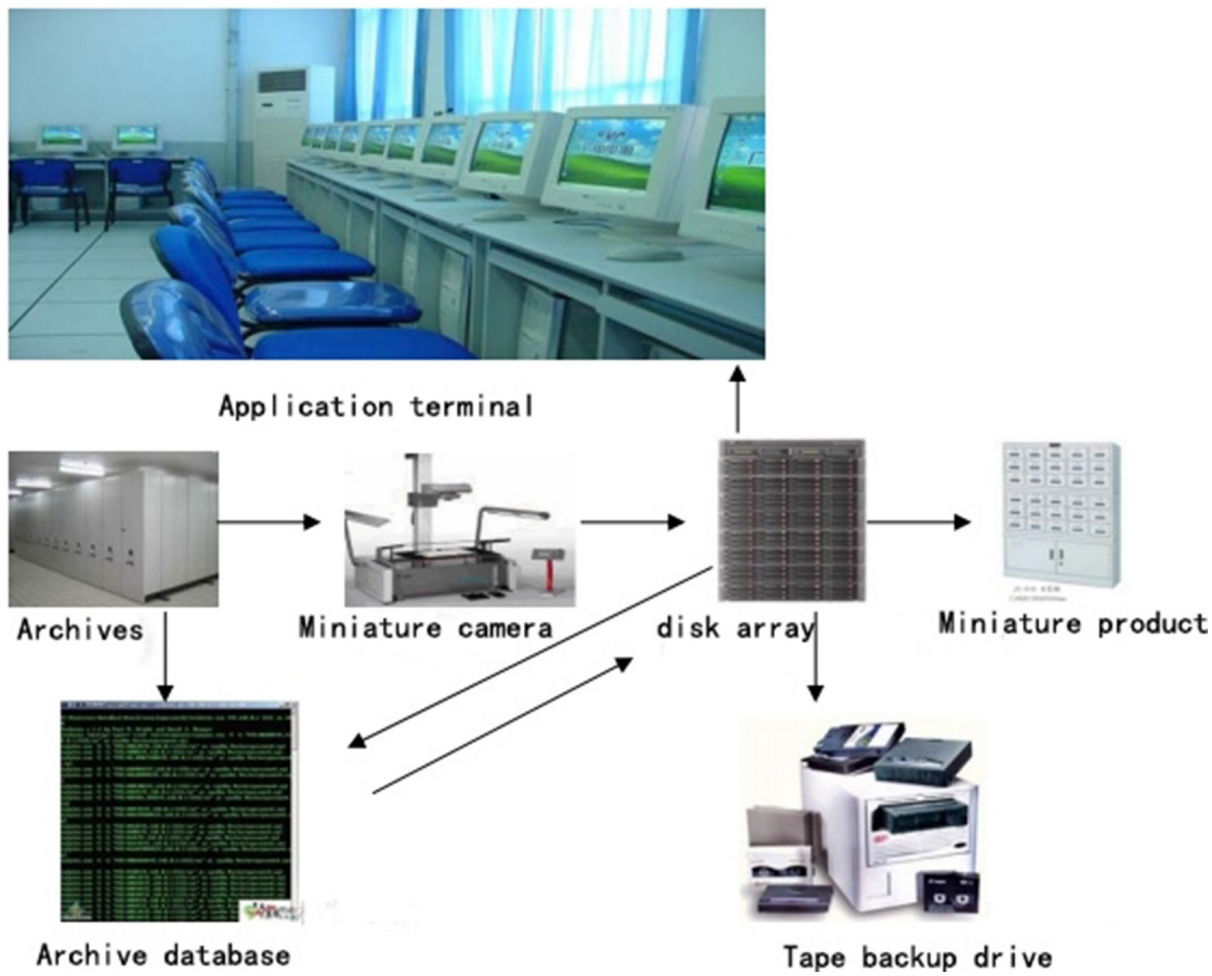
Hardware configuration structure of digital management system based on micro digital image technology.

There was a shortage of funds for the work, and the professional level and awareness of managers still needs improvement. See Figure 2 for the hardware configuration structure of a digital management system based on micro digital image technology.

## 4. Discussion

### 4.1. Advantages of a digital medical record management system based on micro digital imaging technology

Traditional medical record management has obvious disadvantages: (1) the greater complexity of consulting and borrowing medical records, (2) the risk of potentially losing medical records, (3) the large storage space required, and (4) the potential for medical records to be damaged because of various factors (11).

A digital management system based on micro digital image technology has obvious advantages: (1) it can effectively reduce the risks of medical record storage by using the digital imager to scan the medical record once and simultaneously obtain a micro image and a digital image; (2) it can enable electronic management of medical records, avoid the problem of poor quality of medical records after scanning, and reduce the cost of digital reproduction; (3) through the watermark encryption of the scanned medical record data, it can effectively prevent the data from being altered and forged and strengthen the security of managing medical records; and (4) through the establishment of a digital medical record management quality control team, any errors generated in the scanning process may be corrected in time to ensure the stored data are safe and error free (12). Some domestic studies reported that introducing this technology successfully solved difficult problems in medical record management and achieved comprehensive benefits, including saving costs and time, ensuring safety, refining service quality, and improving working efficiency (13). This shows that micro digital imaging technology is an efficient means of medical record processing.

### 4.2. Establishment of a digital management system based on micro digital imaging technology to improve the management of medical records of patients with cerebral hemorrhage

Patients with cerebral hemorrhage tend to be older and have comorbidities. Implementing a digital management system enables the sharing of medical records. This helps to prompt medical personnel to improve primary prevention and monitor and supervise high-risk groups, reducing the incidence of cerebral hemorrhage (14). Establishing a medical record management process for patients with cerebral hemorrhage based on micro digital imaging technology improves medical record management. It helps clinicians to effectively integrate and retrieve the medical record information of their patients, improves its management and efficiency, and optimizes the medical care by using the “4V” characteristics of big data: large amount, high speed, diversity, and value. It is conducive to comprehending a patient's condition and adjusting the treatment plan promptly, digitalising information communication, integrating resources, and comprehensively protecting the rights and interests of patients with cerebral hemorrhage.

### 4.3. Problems with the digital management of medical records of patients with cerebral hemorrhage based on micro digital imaging technology

Implementing a digital management system based on micro digital image technology for the management of medical records of patients with cerebral hemorrhage has a definite effect, but there were still problems and disadvantages.

Managers' awareness of digital management can be relatively poor. Because implementing a digital system for medical record management started late, managers ignored it. In addition, a lack of funds led to a poor foundation for developing the system, which affected its rapid and stable development. The professional level of management was also limited, especially regarding computer operation skills.

Therefore, when implementing a digital management system based on micro digital image technology, it is necessary to achieve these goals: (1) improve understanding of the digital management system, (2) increase investment to improve the infrastructure construction, (3) improve the professional skills and experience of management personnel, (4) attract high-skilled digital talent; and (5) actively promote the use of the system in the medical record management of patients with cerebral hemorrhage.

The biggest disadvantage of this study is that it only explored the medical records of patients with cerebral hemorrhage in the past 3 years, and the effects of applying this technology in other hospitals or to patients with other diseases are unknown. A future study could explore the medical record management of micro digital imaging technology in many hospitals and all diseases.

## 5. Conclusion

The storage of medical records is the foundation, and utilization is the purpose. Establishing a digital management system based on micro digital imaging technology can help realize the effective preservation and utilization of medical records for patients with cerebral hemorrhage. It improves information management, including data sharing, security, and the overall level of information. This is worth promoting.

## Data availability statement

The original contributions presented in the study are included in the article/supplementary material, further inquiries can be directed to the corresponding author.

## Ethics statement

The studies involving human participants were reviewed and approved by Ethics Committee of Western Hospital of Beijing Chaoyang Hospital Affiliated to Capital Medical University. The patients/participants provided their written informed consent to participate in this study.

## Author contributions

X-DL and QZ: conception and design, administrative support, provision of study materials or patients, collection and assembly of data, data analysis and interpretation, manuscript writing, and final approval of manuscript. All authors contributed to the article and approved the submitted version.
